# HIV viral load testing coverage and timeliness after implementation of the wellness anniversary in a paediatric and adolescent HIV clinic in KwaZulu-Natal, South Africa

**DOI:** 10.4102/sajhivmed.v21i1.1016

**Published:** 2020-02-03

**Authors:** Sibusiso E. Kubheka, Moherndran Archary, Kevindra K. Naidu

**Affiliations:** 1 Department of Paediatrics and Child Health, College of Health Sciences, School of Clinical Medicine, University of KwaZulu-Natal, Durban, South Africa; 2 Maternal, Child and Adolescent Health, School of Public Health, University of the Witwatersrand, Johannesburg, South Africa

**Keywords:** HIV, Viral Load Monitoring, Children, Paediatrics, Infectious Diseases

## Abstract

**Background:**

The UNAIDS 2020 Global strategy to reduce the transmission of HIV includes ensuring HIV viral load (VL) testing coverage of at least 90% on all patients on antiretroviral therapy (ART). Routine VL monitoring has been shown to result in earlier detection of treatment failure, timely regimen switches, promotion of adherence to treatment and improved survival. We wanted to assess the introduction of the wellness anniversary in improving routine viral load monitoring.

**Objectives:**

We retrospectively assessed effects of the wellness anniversary on routine VL coverage, timeliness and suppression rates.

**Method:**

The month when the patient initiated ART was designated as the wellness anniversary. On the anniversary month a package of care, which included a routine VL, was delivered. We conducted a retrospective chart audit to assess VL coverage and timeliness between two time periods, from January 2016 to December 2016 (pre-implementation) and from January 2017 to December 2017 (post-implementation).

**Results:**

Timeliness of VL testing improved from 27.5% in the pre-implementation cohort to 49.7% in the post-implementation cohort. Our study showed high VL testing coverage before the implementation of the wellness anniversary with an average of 98.3% VL. There was a significant correlation between timeliness and VL suppression (VLS) in the post-implementation group.

**Conclusion:**

Implementation of the wellness anniversary may improve timeliness of routine VL testing in settings with high VL coverage. Studies looking at the effect of timeliness on VLS and clinical outcomes are needed.

## Introduction

South Africa has the largest HIV epidemic in the world, with about 7.2 million people living with HIV in 2017.^1^ According to the 2016 UNAIDS report, about 320 000 children below 15 years were living with HIV in South Africa in 2016.^2^ While about 90% of people in South Africa living with HIV knew their status, only 59% of children living with HIV in South Africa had access to antiretroviral therapy (ART). In 2016, approximately 78% of people on ART in South Africa were virally suppressed, which translates into about 47% of all people living with HIV.^1,2^

The UNAIDS 2020 Global strategy to reduce the transmission of HIV includes ensuring HIV viral load (VL) testing coverage of at least 90% on all patients on ART.^3^ Routine HIV VL monitoring has been shown to improve earlier detection of treatment failure, timely regimen switches, promoting adherence to treatment and survival.^4^ However, the effect of timeliness of routine VL monitoring on the positive effects of routine VL monitoring is unknown. The World Health Organization (WHO) guidelines from 2013 recommended HIV VL monitoring as a preferred method to monitor treatment response.^5^ In South Africa, routine VL monitoring was rolled out in 2004. Testing intervals are currently at 6 months, 12 months and every 12 months thereafter if patients are suppressed. Routine HIV VL coverage in patients on ART in South Africa was approximately 72% prior to the implementation of the WHO guidelines and 75% by 2015, with 78% VL suppression (VLS).^6^ Factors that were found to contribute to low HIV VL testing coverage in low- and middle-income countries (LMIC) included poor adherence to WHO guidelines, low levels of staff training and lack of funding.^7^ However, the challenges preventing increased HIV VL coverage in South Africa may be different compared to most Sub-Saharan African countries. Quality improvement projects (QIP) have the potential to improve HIV VL coverage, such as QIP reported by Kekana et al., although the scalability of these interventions may be limited.^8^ Sunpath et al. also showed increased VL completion after the implementation of the VL champion in low- and high-traffic ARV clinics.^9^ Improved HIV VL coverage together with active tracing has been implemented in resource-poor settings and has shown to lead towards better clinical outcomes and increased retention in care.^10^

Viral load suppression in children from LMIC is lower when compared to adults in LMIC and children in high-income countries.^11^ HIV VLS has been shown to reduce HIV transmission and improve life expectancy in people living with HIV.^12^ Early VLS in children is associated with better growth, immune and viral responses and psychosocial outcomes.^13^ Good self-reported adherence has been associated with engaged and motivated caregivers, disclosure of child and caregiver status, knowledge of ART and good patient–caregiver–healthcare provider relationships, which result in better outcomes.^14^ Interventions that have been shown to enhance adherence include directly observed therapy by family members, personalised treatment plans, medication diaries, convenient and pleasant clinic appointments and community-based adherence support.^15,16^ Decentralised models of ART delivery may also lead to improved virologic outcomes.^17^ The effects of timely VL testing on treatment outcomes is unknown.

There are multiple measures in place at the clinic to improve adherence to treatment and ensure timely VL testing including active tracing of defaulters, peer-led support groups and WhatsApp groups supervised by social workers. We implemented the wellness anniversary to ensure delivery of an annual standardised holistic package of care. We aimed to assess the effectiveness of the wellness anniversary in improving VL testing coverage and timeliness of testing.

## Methods

### Study design

We conducted a retrospective comprehensive chart audit to assess two time periods, from January 2016 to December 2016 (pre-implementation) and from January 2017 to December 2017 (post-implementation). The primary objective was to assess and compare the coverage and timeliness of HIV VL testing between the two time periods. Secondary objectives were to describe and assess the association of VL testing coverage and timeliness with patient demographics, and to describe and assess the association of VL testing coverage and timeliness with short-term treatment outcomes.

### Study setting

The study was conducted in King Edward VIII Hospital (KEH), Philani Family Clinic, which is a combined adult and paediatric HIV clinic in an urban area. The clinic covers a wide area which includes peri-urban and urban areas. The clinic offers both general paediatric HIV treatment services and tertiary services. The general services are provided by one permanent medical officer, a part-time medical officer and a rotating medical intern, while the tertiary service is provided by the paediatric infectious disease (ID) team once a week. All routine HIV-infected children and adolescents are initiated and followed up by the general service. The tertiary service is a referral clinic for both the general clinic and a large part of KwaZulu-Natal province. The patients managed by the ID team are referred back to the general clinic or the referring hospital with resolution of the referring problem. The audit largely focuses on the patients attending chronic follow-up in the general clinic.

### Study population

The study was conducted on all children below 19 years who attend the paediatric and adolescent HIV clinic in King Edward Hospital. We excluded children who were lost to follow up, children who were transferred out to other ART sites, children who died in the pre-implementation period and children who turned 19 years in the post-implementation period.

### Study sample

The study sample included all the patients who were attending the HIV clinic and met the inclusion and/or exclusion criteria during the two study periods.

### Intervention

We implemented a QIP at KEH in June 2016 to improve the quality of care in our patients on ART. This project involved introducing the concept of ‘wellness anniversary’ for patients and healthcare workers, to use the month of ART initiation as an annual reminder to have their VL test taken as part of a holistic package of annual assessments which included safety laboratory tests, weight, height and developmental screening. Prior to this, the clinic implemented a VL register to track VLs performed and to follow up results on a weekly basis. The effectiveness of the VL register was not assessed. The project was discussed with the management, nursing staff, counsellors and social workers working at the clinic to ensure commitment from the clinic team. Maternal Adolescent and Child Health (MatCH), a non-governmental organisation, provided the colour-coded anniversary month stickers. The nurses at the weighing station educated the patients on the aims of the wellness anniversary month. They identified the anniversary month and attached the stickers on the outpatient file and the ART clinic file. The patient was also informed about when their anniversary month was. The patients who had a VL done more than 6 months before the anniversary month and were suppressed received the package of care on their anniversary month. The patients who were not suppressed were tested according to the guideline for treatment failure. The implementation was ongoing as new patients were initiated on ART during the study period.

### Data collection

A list of all the participants and their folder numbers was generated from the Tier.net database. Files were retrieved from the filing room according to the list, file audits were performed, and data were entered in the data collection Microsoft Excel spreadsheet. Duplication of patients’ records, patients transferred out to other sites, patients lost to follow up, and deceased patients were removed from the database.

HIV VL testing was assessed by presence of results on the results sheet in the patient’s chart or the result log in the clinical chart or a specimen lab sticker in the file, or the note of the doctor’s order for the test. VL results that were not in the patient’s files were traced on the National Health Laboratory Services Trakcare system. Absolute VL results were recorded to assess the outcomes. VLs results were categorised as follows: ‘lower than detectable level’ (LTDL) if less than 50 copies per millilitre (cp/mL), ‘50–999 cp/mL’, ‘1000 cp/mL and above’, ‘None’ if no VL was done in the audit period, and ‘Not Applicable’ if a VL was not due either because the patient is on a holding regimen or has recently initiated ART and 6 months on treatment has not passed by the end of the audit.

### Outcomes and measurements

Viral load coverage was assessed by the proportion of patients with at least one routine VL testing performed in the 12 months within the study period. Timeliness was described by how promptly the patient received testing once due for testing (i.e. 12 months after the prior VL). We considered VLs which were done a month before, on or after the wellness anniversary as timely. The analysis for timeliness was performed on patients with 2 VLs or performed in a 12-month period as patients needed a maximum of two VLs in a period of 12 months as per the testing guidelines. Patients with more than two VLs were likely to have treatment failure and more frequent VL testing. Short-term clinical outcomes were assessed by HIV VLS.

### Statistical analysis

Statistical analysis was done by looking at means, medians and standard deviations. Measures for statistical significance used were Pearson’s chi squared test and *p*-values.

### Bias

All patients were included for file review to reduce selection bias. There was no blinding to reduce bias when files were reviewed.

### Ethical consideration

Ethics approval was obtained from the Biostatistics Research Council (BREC) of the University of KwaZulu-Natal (approval number BE624/18). Gatekeeper approval was obtained from King Edward VIII Hospital and the Department of Health.

## Results

We reviewed 1468 patient folders. The pre-implementation dataset excluded 387 patients that were lost to follow-up, 209 patients that were transferred out and 24 patients that died. The final number of patients included for analysis was 850 in the pre-implementation group. The post-implementation dataset excluded 38 patients that turned 19 before the end of the audit, 24 patients that were lost to follow-up and 23 patients that were transferred out. We ended up with 765 patients in the post-implementation group.

### Baseline characteristics

Table 1 shows baseline characteristics of the two groups. The pre-implementation group had 464 males and 386 females. The average age was 10.9 years, and the median age was 10.9 years with a standard deviation of 4.6. The post-implementation group had 422 males and 343 females. The average age was 11.9 years, and the median age was 12.1 years with a standard deviation of 4.39. Age was further categorised into groups for analysis.

**TABLE 1:** Baseline characteristics.

Baseline characteristics	Pre-implementation	Post-implementation
**Participants (*n*)**	850	765
**Age (*n*)**		
0 to < 3 years	47	16
3 to < 10 years	303	231
10 to < 19 years	500	518
**Gender (*n*)**		
Male	464	422
Female	386	343
**Viral loads (*n*)**		
None	14	16
LTDL	561	452
50–399	126	123
400–999	24	36
> 1000	111	113
Unsuitable	11	22
Not applicable	3	3

LTDL, lower than detectable level.

### Viral load coverage

The baseline VL coverage was 98.3% in the pre-implementation group and 97.8% in the post-implementation group. There was 100% coverage in the 0–3-year age group in the post-implementation audit (Table 2).

**TABLE 2:** Viral load suppression rate by age groups.

VL suppression rate (%)	Pre-implementation	Post-implementation
**By age**		
Overall	86.5	84.4
0 to < 3 years	56.8	69.0
3 to < 10 years	87.7	85.7
10 to < 19 years	88.0	84.2

VL, viral load.

### Viral load suppression rate

Viral load suppression was 86.5% in the pre-implementation group and 84.4% in the post-implementation group. The suppression rates in the 0–3-year age group were distinctly lower with 56.8% VLS in the pre-implementation group and 69% post-implementation. Suppression rates in the > 3–10 years were 87.7% and 85.7% in the pre- and post-implementation groups, respectively.

### Viral load timeliness

**FIGURE 1:**
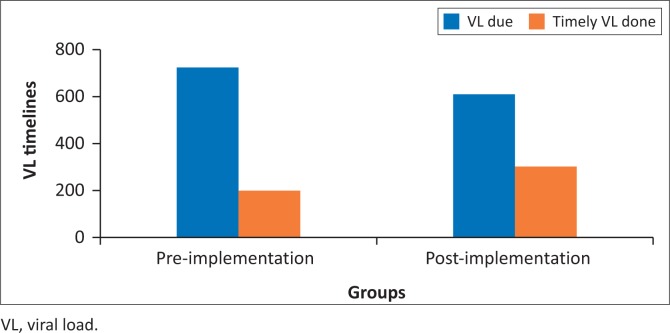
Overall timeliness in pre- and post-implementation groups.

Viral load timeliness was 27.5% in the pre-implementation group and 49.7% in the post-implementation group (Figure 1). There were 164 timely VLs in the pre-implementation group, of which 130 were suppressed, producing a VLS rate of 79%. There was a total of 341 timely VLs done post-implementation, out of a total of 723 VLs, and of those only 37 were unsuppressed, producing a VLS rate of 89% in that post-implementation group. There was a statistically significant association between timeliness of VL testing and VLS after implementation of the wellness anniversary with a Pearson’s chi square of 11.18 and *p*-value of 0.001 (Table 3).

**TABLE 3:** Timely viral load versus viral load suppression in the post-implementation group.

Post-implementation	Timely VL	Not timely VL	Cumulative
**Suppressed**			
Number	304	306	610
Percentage	49.8	50.2	100
**Not suppressed**			
Number	37	76	113
Percentage	32.7	67.3	100

VL, viral load.

Pearson’s chi square (1) = 11.1776; *p* = 0.001.

## Discussion

Our study showed a high VL testing coverage at this urban paediatric HIV clinic before implementation of the wellness anniversary with an average of 98.3% VL coverage pre-implementation of the wellness anniversary. This was attributed to an already existing system of tracking all the patients that are due for VL testing, as well as defaulters who have not returned for their results.

Routine VL monitoring has been associated with better clinical outcomes.^4^ The wellness anniversary intervention did not have a significant positive or negative effect on the VL coverage in the clinic, with a post implementation overall coverage of 97.8%. This may be attributed to high baseline VL coverage. The patients who did not have VLs done were mostly patients with anniversary months in December or January when patients return to family homes outside of the city during the holidays.

Timeliness of VL tests was low in our paediatric and adolescent patients with an average of 27.5% done timeously before implementation. This improved to 49.7% in patients who had suppressed VLs after the implementation of the wellness anniversary. This analysis was only on patients with VLs below 1000. There was particularly low timeliness in January and December in both groups which we have attributed to the holiday season. During this period the pharmacy dispenses 2 months’ supply of treatment for patients with good adherence and treatment response. Some patients also go on holiday during this period. The timeliness of VL testing was expected to correlate with early detection of treatment failure and timely intervention which should lead to higher virological suppression rates and less drug resistance. However, the results did not show this, as overall suppression rates did not improve.

The overall VLS rate was above 80% in the pre-implementation group, which is higher than the cascaded target of 72.9% set by the UNAIDS 2020 strategy. There was no overall improvement in suppression rates in our study. The 0–3 year age group had a particularly low suppression rate and improved after implementation. Time to suppression in this group may be affected by factors such as high baseline VL, poor tolerability of syrups and caregiver-related issues.^18^ Maternal ART and early infant diagnosis are likely to improve this, with more infants with lower VLs at ART initiation and earlier VLS. There was a statistically significant increase in VLS in the patients who had timely VLs in the post-implementation group. This may be attributed to the fact that patients with suppressed VLs only need 12 monthly VLs, which may be the bulk of patients who subsequently had timely VLs. Further, patients may have been better able to anticipate the date of the VL testing during the implementation and may have re-enforced adherence resulting in improved VLS. Studies looking at regimen switches and re-suppression as a result of timely VL testing may be more conclusive to show the effect of timely VL testing.

Although our study consisted of a comprehensive chart review of a large cohort of patients, limitations include that we did not record ART regimens the patients were on and whether there was an increase in ART regimen switches with the timely VL testing. Implementation of the wellness anniversary had both patient-related and staff-related obstacles, which may be overcome easily even in LMIC setting ART clinics. Patients were initially concerned with the possible stigma expressed by other patients because of a visible sticker on their clinical charts, which required a change in the position of the sticker so that it was less visible. The process of addressing concerns also helped with patient engagement and ownership of their treatment monitoring which may contribute to retention-in-care. Once the initial phase of rolling out the wellness anniversary was completed, the responsibility of assigning the wellness anniversary and putting the stickers on the files was assigned to the doctors. Patients that were transferred from other institutions often did not have detailed transfer letters, and patients could not recall the month they commenced ART. The month of transfer ended up being their wellness anniversary. We would recommend that assigning the wellness anniversary should also be flexible to accommodate the patient’s and caregiver’s lifestyles such as avoiding routine monitoring in school-going children during school holiday months. We feel that these findings are generalisable to other large paediatric and adolescent clinics and offer a low-cost strategy to improve the VL testing.

## Conclusion

The implementation of the wellness anniversary in the setting of a clinic with a high VL coverage may improve timeliness of VL tests done. Further studies are needed to assess the effects of timely VLs on VLS and clinical outcomes.
